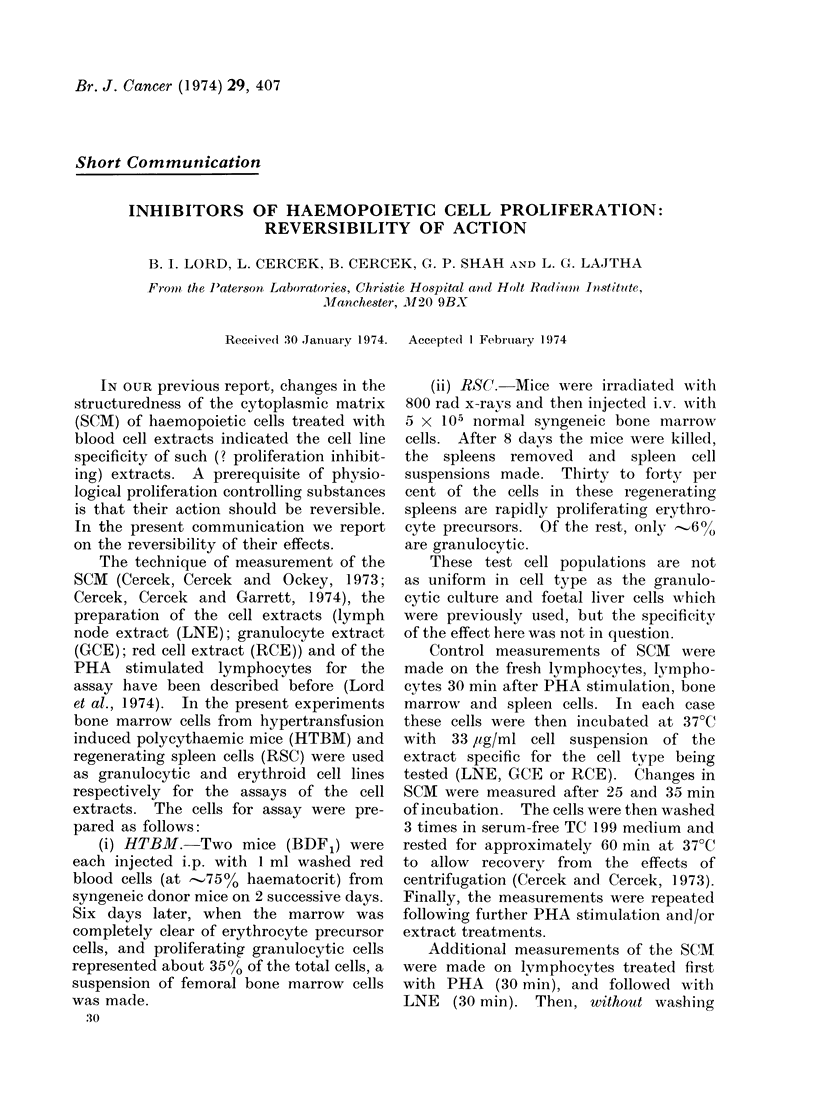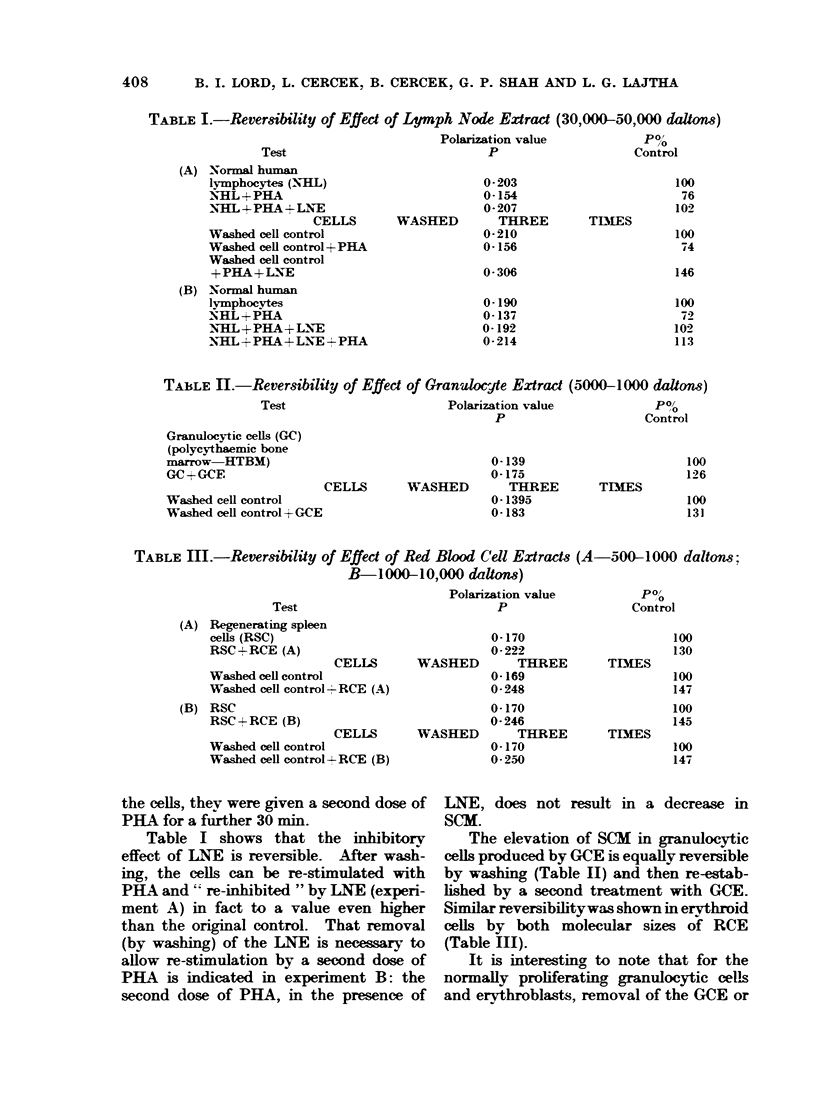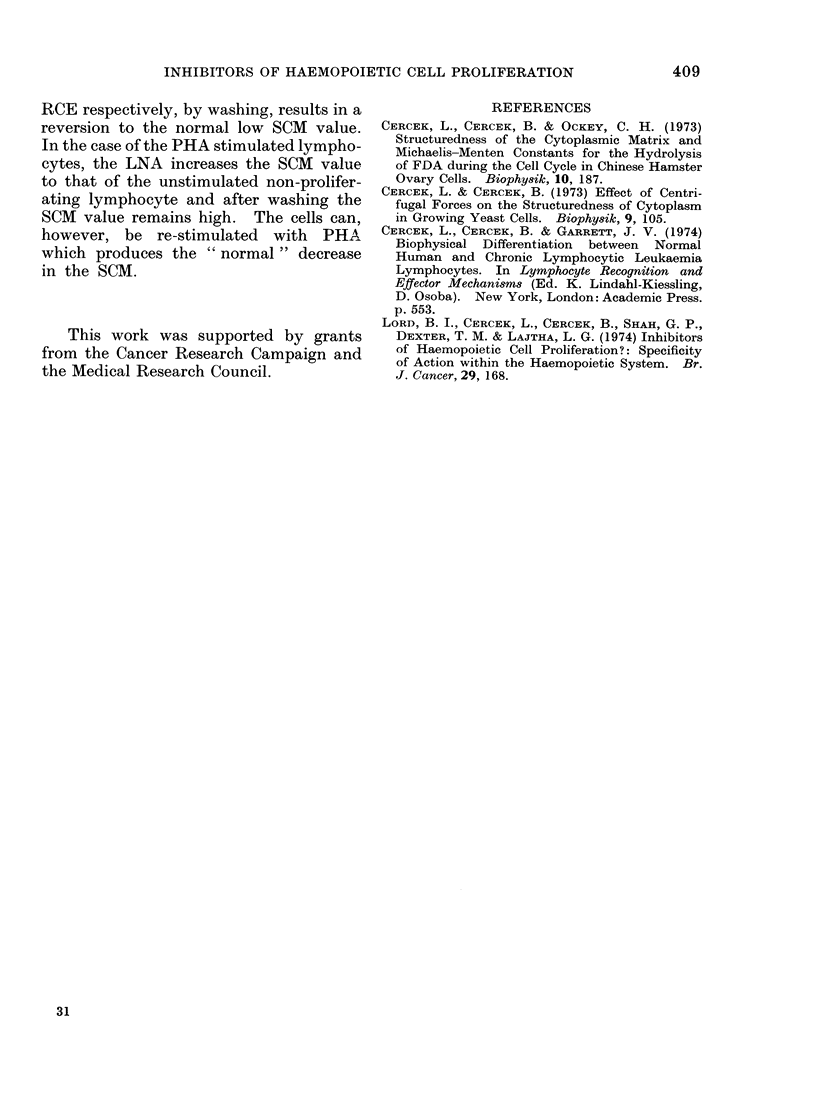# Inhibitors of haemopoietic cell proliferation: reversibility of action.

**DOI:** 10.1038/bjc.1974.89

**Published:** 1974-05

**Authors:** B. I. Lord, L. Cercek, B. Cercek, G. P. Shah, L. G. Lajtha


*Br. J. Cancer* (1974) **29**, 407

***Short Communication***

### INHIBITORS OF HAEMOPOIETIC CELL PROLIFERATION: REVERSIBILITY OF ACTION

B. I. LORD, L. CERCEK, B. CERCEK, G. P. SHAH AND L. G. LAJTHA

*From the Paterson Laboratories, Christie Hospital and Holt Radium Institute, Manchester, M20 9BX*

Received 30 January 1974. Accepted 1 February 1974

In our previous report, changes in the structuredness of the cytoplasmic matrix (SCM) of haemopoietic cells treated with blood cell extracts indicated the cell line specificity of such (? proliferation inhibiting) extracts. A prerequisite of physiological proliferation controlling substances is that their action should be reversible. In the present communication we report on the reversibility of their effects.

The technique of measurement of the SCM (Cercek, Cercek and Ockey, 1973; Cercek, Cercek and Garrett, 1974), the preparation of the cell extracts (lymph node extract (LNE); granulocyte extract (GCE); red cell extract (RCE)) and of the PHA stimulated lymphocytes for the assay have been described before (Lord *et al.*, 1974). In the present experiments bone marrow cells from hypertransfusion induced polycythaemic mice (HTBM) and regenerating spleen cells (RSC) were used as granulocytic and erythroid cell lines respectively for the assays of the cell extracts. The cells for assay were prepared as follows:

(i) *HTBM*.—Two mice ($BDF_1$) were each injected i.p. with 1 ml washed red blood cells (at ~75% haematocrit) from syngeneic donor mice on 2 successive days. Six days later, when the marrow was completely clear of erythrocyte precursor cells, and proliferating granulocytic cells represented about 35% of the total cells, a suspension of femoral bone marrow cells was made.

(ii) *RSC*.—Mice were irradiated with 800 rad x-rays and then injected i.v. with $5 \times 10^5$ normal syngeneic bone marrow cells. After 8 days the mice were killed, the spleens removed and spleen cell suspensions made. Thirty to forty per cent of the cells in these regenerating spleens are rapidly proliferating erythrocyte precursors. Of the rest, only ~6% are granulocytic.

These test cell populations are not as uniform in cell type as the granulocytic culture and foetal liver cells which were previously used, but the specificity of the effect here was not in question.

Control measurements of SCM were made on the fresh lymphocytes, lymphocytes 30 min after PHA stimulation, bone marrow and spleen cells. In each case these cells were then incubated at 37°C with 33 μg/ml cell suspension of the extract specific for the cell type being tested (LNE, GCE or RCE). Changes in SCM were measured after 25 and 35 min of incubation. The cells were then washed 3 times in serum-free TC 199 medium and rested for approximately 60 min at 37°C to allow recovery from the effects of centrifugation (Cercek and Cercek, 1973). Finally, the measurements were repeated following further PHA stimulation and/or extract treatments.

Additional measurements of the SCM were made on lymphocytes treated first with PHA (30 min), and followed with LNE (30 min). Then, *without* washing

TABLE I.—*Reversibility of Effect of Lymph Node Extract* (30,000–50,000 *daltons*)

| | Test | Polarization value $P$ | $P\%$ Control |
|---|---|---|---|
| (A) | Normal human lymphocytes (NHL) | 0·203 | 100 |
| | NHL+PHA | 0·154 | 76 |
| | NHL+PHA+LNE | 0·207 | 102 |
| | CELLS WASHED THREE TIMES | | |
| | Washed cell control | 0·210 | 100 |
| | Washed cell control+PHA | 0·156 | 74 |
| | Washed cell control +PHA+LNE | 0·306 | 146 |
| (B) | Normal human lymphocytes | 0·190 | 100 |
| | NHL+PHA | 0·137 | 72 |
| | NHL+PHA+LNE | 0·192 | 102 |
| | NHL+PHA+LNE+PHA | 0·214 | 113 |

TABLE II.—*Reversibility of Effect of Granulocyte Extract* (5000–1000 *daltons*)

| Test | Polarization value $P$ | $P\%$ Control |
|---|---|---|
| Granulocytic cells (GC) (polycythaemic bone marrow—HTBM) | 0·139 | 100 |
| GC+GCE | 0·175 | 126 |
| CELLS WASHED THREE TIMES | | |
| Washed cell control | 0·1395 | 100 |
| Washed cell control+GCE | 0·183 | 131 |

TABLE III.—*Reversibility of Effect of Red Blood Cell Extracts* (*A*—500–1000 *daltons*; *B*—1000–10,000 *daltons*)

| | Test | Polarization value $P$ | $P\%$ Control |
|---|---|---|---|
| (A) | Regenerating spleen cells (RSC) | 0·170 | 100 |
| | RSC+RCE (A) | 0·222 | 130 |
| | CELLS WASHED THREE TIMES | | |
| | Washed cell control | 0·169 | 100 |
| | Washed cell control+RCE (A) | 0·248 | 147 |
| (B) | RSC | 0·170 | 100 |
| | RSC+RCE (B) | 0·246 | 145 |
| | CELLS WASHED THREE TIMES | | |
| | Washed cell control | 0·170 | 100 |
| | Washed cell control+RCE (B) | 0·250 | 147 |

the cells, they were given a second dose of PHA for a further 30 min.

Table I shows that the inhibitory effect of LNE is reversible. After washing, the cells can be re-stimulated with PHA and " re-inhibited " by LNE (experiment A) in fact to a value even higher than the original control. That removal (by washing) of the LNE is necessary to allow re-stimulation by a second dose of PHA is indicated in experiment B: the second dose of PHA, in the presence of LNE, does not result in a decrease in SCM.

The elevation of SCM in granulocytic cells produced by GCE is equally reversible by washing (Table II) and then re-established by a second treatment with GCE. Similar reversibility was shown in erythroid cells by both molecular sizes of RCE (Table III).

It is interesting to note that for the normally proliferating granulocytic cells and erythroblasts, removal of the GCE or

RCE respectively, by washing, results in a reversion to the normal low SCM value. In the case of the PHA stimulated lymphocytes, the LNA increases the SCM value to that of the unstimulated non-proliferating lymphocyte and after washing the SCM value remains high. The cells can, however, be re-stimulated with PHA which produces the " normal " decrease in the SCM.

This work was supported by grants from the Cancer Research Campaign and the Medical Research Council.

REFERENCES

CERCEK, L., CERCEK, B. & OCKEY, C. H. (1973) Structuredness of the Cytoplasmic Matrix and Michaelis–Menten Constants for the Hydrolysis of FDA during the Cell Cycle in Chinese Hamster Ovary Cells. *Biophysik*, **10**, 187.

CERCEK, L. & CERCEK, B. (1973) Effect of Centrifugal Forces on the Structuredness of Cytoplasm in Growing Yeast Cells. *Biophysik*, **9**, 105.

CERCEK, L., CERCEK, B. & GARRETT, J. V. (1974) Biophysical Differentiation between Normal Human and Chronic Lymphocytic Leukaemia Lymphocytes. In *Lymphocyte Recognition and Effector Mechanisms* (Ed. K. Lindahl-Kiessling, D. Osoba). New York, London: Academic Press. p. 553.

LORD, B. I., CERCEK, L., CERCEK, B., SHAH, G. P., DEXTER, T. M. & LAJTHA, L. G. (1974) Inhibitors of Haemopoietic Cell Proliferation?: Specificity of Action within the Haemopoietic System. *Br. J. Cancer*, **29**, 168.